# Task shifting for musculoskeletal disorders in Norwegian primary care: a qualitative interview study of general practitioners and specialist musculoskeletal physiotherapists

**DOI:** 10.1080/02813432.2024.2384043

**Published:** 2024-08-02

**Authors:** Kenneth Chance-Larsen, Michael Backhouse, Richard Collier, Tobba Sudmann

**Affiliations:** a Western Norway University of Applied Sciences, Bergen, Norway; b Warwick Clinical Trials Unit, University of Warwick, Warwick, UK; c School of Allied Health and Nursing, University of Chichester, Chichester, UK

**Keywords:** General practice, multidisciplinary collaboration, musculoskeletal, physiotherapy, primary care, skill-mix, task shifting

## Abstract

**Objective:**

To explore the views of general practitioners and physiotherapists on the current model of care for patients with musculoskeletal disorders in Norwegian primary care, and if the English First Contact Practitioner model, where patients have access to multiple professional groups with musculoskeletal health expertise, could inform service development.

**Design, setting, and subjects:**

We analysed interviews with five GPs and 11 physiotherapists and used Lipsky’s theories about street-level bureaucracy and Foucault’s theories of mechanisms of power and institutional structures to explore task shifting and cooperation between different professions.

**Results and interpretation:**

The empirical material reflected a multi-faceted discourse about skill-mix in primary care, where financial factors, perceptions about competence, and task preferences moderated attitudes to task shifting. Competition and cooperation coexist between the professions, and the seemingly gradual blurring between historical hegemony and new models of care creates both alliances and rivalries. Examples of deviations from the Choosing Wisely principles and evidence-based practice indicate that both general practitioners and physiotherapists balance the roles of patient advocate, gatekeeper, and *homo economicus*, in a context where task shifting is challenged by established practice. It appears that the management of patients with musculoskeletal disorders is fragmented and to some extent reflects a supply-driven system.

## KEYPOINTS

- The demand on primary care is placing increasing pressure on general practitioners.
- Multidisciplinary teamwork has potential to improve primary care, for both healthcare professionals and for patients.
- In this study, it appeared that both competition and cooperation exist between general practitioners and physiotherapists around the management of patients with musculoskeletal disorders in primary care.
- There is a case for change in service delivery for patients with musculoskeletal disorders in Norwegian primary care.

## Introduction

General Practitioners (GPs) in Norway have seen an increase in workload since the GP system was established in 2001, despite a growth in the number of GPs and a reduction in the average number of patients on GPs lists [1]. The growth in non-communicable diseases and increasing numbers of individuals living with multiple long-term conditions is placing growing pressure on primary care systems [2] and the Norwegian Association of GPs have suggested that an additional 1,000 GPs are needed [3]. Multimorbidity tends to increase with age, and the predicted demography shift towards population aging in Norway is anticipated to contribute to increased burden of disease and use of healthcare services over the coming decades [4]. Musculoskeletal disorders account for the largest proportion of Years Lived with Disability (YLD) in Norway and is the third largest cause of Disability Adjusted Life Years (DALY) [5]. During 2019, 32% of Norwegians accessed a GP practice with a musculoskeletal disorder, at an estimated societal cost of more than 255 billion NOK per year [5]. This context presents a fundamental challenge to the established model of primary care in Norway and offers an opportunity to explore new ways of collaborating and structuring services for people with musculoskeletal disorders.

Task shifting is prominent in the recent report on the Norwegian GP service, and physiotherapy is highlighted as a profession that provides expertise that can extend beyond that of GPs in musculoskeletal practice [4]. Broadening the skill-mix within a team and task shifting between professions can give increased flexibility and efficiency, and lead to a better use of finite resources [4,6]. To date, multidisciplinary team working is less developed in Norwegian primary care practices compared to many countries [4,7]. It is therefore of interest to explore whether the relative monopoly of GPs in Norwegian primary care can be linked to its organisation and financial model, and if this presents unintentional barriers to meaningful task sharing between healthcare professional groups. Users of primary care have asked for more multidisciplinary cooperation as well as better integration of the GP service with other health and social care services [4].

The multi-professional First Contact Practice model of care was introduced in England to enhance care and increase capacity in primary care and allows a patient with a musculoskeletal disorder to see a physiotherapist instead of their GP at the GP surgery [8]. Physiotherapists in the musculoskeletal First Contact Practice role are working at the top of their clinical scope of practice and can assess, diagnose, treat, and discharge patients independently without the need for a medical referral, with the intention of providing patients with faster access to the right care. Research into the effects of musculoskeletal First Contact Practice physiotherapists indicates improved clinical results, reduced prescribing and use of imaging, more appropriate onward referrals, higher surgical conversion rates, and high patient satisfaction scores [8–11]. However, there are also reported challenges associated with the role, manifest as uncertainty amongst musculoskeletal First Contact Practitioners about the lack of role clarity, the burden of responsibility, unpreparedness for the primary care environment, and burnout 12. From an economic perspective task shifting from GPs to musculoskeletal First Contact Practitioners has been shown to reduce the cost of providing healthcare, and the NHS Long Term Workforce Plan sets out the commitment make national funding available to support every general practice to have a musculoskeletal First Contact Practitioner by 2032/33 13.

In this study we explored the views of GPs and physiotherapists on the current model of care for patients with musculoskeletal disorders in Norwegian primary care, what is working well and what can be improved. We explored if using an alternative model of care, such as the First Contact Practice model where physiotherapists with specialisation in musculoskeletal health work alongside and are co-located with GPs, would be possible, with the aim to generate insights around the required conditions for possible changes such as task shifting and further development of multidisciplinary team working.

## Materials and methods

We report this study in accordance with the Standards for Reporting Qualitative Research (SRQR) 14. We used a qualitative and exploratory design, drawing on empirical material from individual in-depth semi-structured online video interviews. We depart from a social constructivist theoretical foundation, with an understanding that the development of knowledge is constructed by social processes, shaped by individual interactions, and that is historically and culturally specific based on human perception and social experience [15, 16].

We used a purposive sampling approach through contacting relevant professional organisations and through personal contacts and networks. Potential participants were invited to make an informed decision on whether to participate via email that included a Participant Information Sheet (PIS). A positive reply via email about agreeing to take part in an interview was considered to constitute written informed consent, this was explicit from the PIS. Participants were invited to an online interview (Microsoft Teams) with a member of the research team experienced in qualitative interviewing (KCL). The interviews were conducted in Norwegian.

We developed an interview guide and piloted this ahead of the first interview. The interview guide included questions about structural conditions for musculoskeletal services (i.e. governance, economic, workforce), processes (i.e. task shifting, access, continuity/coordination/comprehensiveness of care), outcomes (i.e. quality/efficiency of care, equity), and organisational (i.e. primary care practices, co-location). The guide was further developed based on completed interviews, an iterative process facilitated by specifically asking participants whether there were any other questions they thought relevant but had not been asked. The interviews were conducted from April to October 2023, they were digitally audio-recorded (duration between 50 and 101 min) and subsequently transcribed verbatim. The transcription process included uploading audio recordings to a secure online transcription service [17] before being checked and corrected by KCL.

The interview transcripts were uploaded to NVivo 12 (QSR International) for management. KCL and TS analysed the empirical material individually and collaboratively. Reflexive field notes were developed during the interviews and transcription process, and these were included in the analytical process. We recognised that the researcher is an integral part of the study, thus exerting influence on the production of empirical material. The interviewer (KCL) has a professional background as a physiotherapist with an MSc degree in Manual Therapy from the UK, he coordinated the development of a national musculoskeletal capabilities framework for First Contact Practitioners [9] and he led a post-graduate MSc module preparing First Contact Practitioners for primary care roles in England. This demands ethical reflexivity and methodological considerations from the researchers. Participants might have shaped their replies based on what they thought the interviewer might want to hear, and on having a shared interest in musculoskeletal disorders. On the other hand, this knowledge about First Contact Practice might have scaffolded the interviews to instill a level of trust. Information about the interviewer’s expertise was only offered when it came up naturally in conversation or when a participant specifically enquired. Follow-up questions were asked to give participants opportunity to share experiences and evaluations, whether these were professional or political. KCL and TS are native Norwegian speakers and therefore have a different relationship to the empirical material than do MB and RC, who were given access to the empirical material through oral and written translations. This means that MB and RC only have access to selected quotations as well as material already interpreted by KCL and TS.

In the meeting between the healthcare professional, the actor of the welfare state, and the citizen, bureaucracy and power come into play in ways that are partly intended and partly unintended. We have used Lipsky’s theories on street-level bureaucracy [18] to analyse the working practices and beliefs of frontline workers in public services and how they enact public policy in their work [19]. We also draw upon a Foucauldian perspective [20] on mechanisms of power and institutional structures. If power is to be understood as access to resources (e.g. knowledge, fiscal, material), it can be used or mobilised for specific aims. These perspectives challenge possible preconceptions held – both on the part of the researchers and participants – about seemingly self-evident truths residing in current thought and value systems, thus providing an alternative view relevant to the field of healthcare professions [21,22]. Foucauldian and constructivist perspectives facilitate discovery of unarticulated views and practices, paradoxes or contradictions between practice, policies, and funding, which in turn inform the focus of our study.

To be able to utilise the theoretical lenses provided by Lipsky and Foucault, we analysed the empirical material using the five methodological principles outlined by Alvesson and Kärreman (defragmentation, defamiliarisation, problematisation, broad scholarship, reflexive critique) [23]. This analytical approach is iterative, critical, and reflexive, counters assumptions about patterns in the empirical material with suggestions that the social reality is more fragmented and complex, and facilitates distance from existing pre-understandings. Using the five principles made it possible to pursue a critical analysis that moved beyond the ‘face-value’ of the interviews, by seeking to understand the kind of processes that made the participants’ statements, interpretations, and practices relevant for them. Central to this approach is a recognition that the research practice is both uncertain and complex, and any interpretation is contestable [24].

Ethical approval (an assessment of the processing of personal data) was granted by SIKT (Norwegian Agency for Shared Services in Education and Research, Reference number 493790).

## Findings and discussion

We have chosen to merge the findings and discussion sections as this allowed us to present emergent findings, theories, and interpretations in the same context, in line with the methodological principles outlined above [23]. We believe merging these sections makes our analytical process more transparent, and justifies our generation of hypotheses and theories to explain the empirical material.

Table 1 shows participant demographics. We recruited 16 participants, five GPs and 11 physiotherapists, who met the inclusion criteria of having experience of working in primary care and with a musculoskeletal caseload. All the GPs were self-employed working under contract with municipalities, 10 of the physiotherapists had contracts with municipalities and one worked non-clinically. Three of the GPs were full-time clinical, two were part-time clinical and part-time academic/research. Seven of the physiotherapists were full-time clinical, two were part-time clinical and part time academic/research, one was part-time clinical and part-time professional body employee, one worked full-time as a professional body employee. Eight of the physiotherapists were licensed musculoskeletal physiotherapists (*manuellterapeut*). A manuellterapeut holds a Masters degree in Manual Therapy and has extended rights, including referral rights to imaging (x-ray, MRI, CT, etc.) and specialist care, and they can issue sick notes for up to 12 weeks. We refer to this group as ‘manual therapists’ hereafter.

**Table 1.** Participant demographics.

	*N (*female)	Year experience (range)	Centrality Index^#^	Municipality contract
GPs	5 (2)	16.6 (8–35)	2	5
Physiotherapists	3 (3)	12.3 (8–30)	3,4,6	3
Manual therapists	8 (2)	30.1 (8–47)	1-4	7

^#^ The Centrality Index classify municipalities from 1 (central) to 6 (rural) based on access to service functions and labour markets.

In the next sections we present our construction of theoretical reflections, under two main subheadings: (1) *Attitudes to task shifting moderated by financial factors, perceptions about competence, and task preferences*, and (2) *Historical hegemony and established practice challenged by the emergence of task shifting*. We describe how the empirical material both inductively and deductively pointed towards an active discourse around task shifting and skill-mix in primary care. We discuss how clinicians take on the multiple roles of patient advocate, health service gatekeeper, and homo economicus – an economic concept where people pursue their material self-interests opportunistically [25] – and how they manage conflicting forces in balancing these. We explore how competition and collaboration seem to co-exist, and how an apparent blurring of lines between historical hegemony and emerging models of care creates both alliances and rivalries. Finally, we examine these reflections within our chosen theoretical framing.

### Attitudes to task shifting moderated by financial factors, perceptions about competence, and task preferences

The participants contributed views that encapsulate nuanced and multifaceted discourses pertaining to task shifting. An engagement with professional narratives about changes to professional boundaries, to regulations, and to political initiatives was evident. Task shifting, multidisciplinary working, and skill-mix in primary care appeared to be important and both GPs and physiotherapists were preoccupied by how these already influence current practice and how they might affect the future evolution of the primary care workforce. Relevant to these preoccupations were financial aspects, perceptions about competence, and task preferences. We will introduce these in turn.

#### Financial aspects

GPs saw professional opportunities related to co-location and closer collaboration with physiotherapists, but also highlighted financial barriers to such a development. The more expensive pension arrangements and comparatively higher salaries of nurses to that of medical secretaries was given as the reason why relatively few GPs employ nurses, an illustration of how the current system of funding prevents a better integration of other professions into the GP service.

No one wants their employees enrolled with KLP [the municipal pension provider for nurses], as they then pay contributions for a long time after retirement. (…) Let’s take a nurse, for example, who handles diabetes control, asthma control and things like that, they can generate a profit for the practice even if they cost more. But as long as you don’t have that specialised function it becomes an expensive undertaking for GP practices. I think it’s getting less and less popular, there were more nurses in the past. But over time (…) health secretary education came about, and it sort of got diluted and they took over those jobs because they were somewhat cheaper. (GP)

Another financial challenge was the inherent financial incentive that exists for many GPs, which creates resistance to establishing multidisciplinary teams:

No, perhaps there’s a kind of a resistance among GPs these days. You’ve got primary care teams, which have been kind of researched and looked at, and it gets stuck both for the financial funding bit, and GPs are put off. Or someone in the group is turned off when they hear that now they’ll be required to create a multidisciplinary team. So there has to be an incentive of sorts behind that, and unfortunately finances have become a very big incentive for GPs. (GP)

A physiotherapist explained how co-location with GPs has developed despite the current financial system:

Co-location is good, but again there are a number of problems with the current solution of being self-employed and having to find our own premises. We have now taken the initiative to move into new premises jointly with the doctors. But it’s coincidental, it’s because we want to do it. (Physiotherapist)

#### Competence

Participants’ perceptions about competence seemed to inform their views on task shifting. Direct access to a manual therapist without the need for a referral has been in place since 2006, and this was introduced in 2018 for physiotherapists. The municipal contract system for physiotherapists is a de facto recognition by regulators that these healthcare professionals have the required competencies to be safe and effective primary contacts for patients with musculoskeletal disorders. When a GP expressed reservations about how well a physiotherapist – as opposed to a manual therapist – could detect red flags (signs of potential serious pathology) when examining a patient, he indirectly questions the change in policy to allow direct access to physiotherapists:

What are red flags, what are yellow flags? When do you need to tune in to these? When do you need to make a phone call to the GP? I think it’s a bit of rapid liberalisation without considering the consequences. I think a manual therapist is trained to detect red flags better than a physiotherapist. (GP)

Both GPs, physiotherapists, and manual therapists raised concerns about the lack of mandatory training for physiotherapists (including manual therapists) to maintain their professional registration and municipality contracts:

After all, there is no requirement for those of us in private practice to undertake further education and updating. We encourage this, but there is no requirement from public bodies for it. There are definitely some people who never attend any courses. (Manual Therapist)

You can only cross your fingers and hope it goes well for each individual [patient], but you know it’s not good enough that someone who hasn’t been on course for 20 years to function as a primary contact. (GP)

The lack of formal continuing professional development requirements for physiotherapists (including manual therapists) highlighted by our participants represents a central challenge to organisational change in Norwegian primary care. Whilst evidence from the United Kingdom indicate many benefits from task shifting from GPs to musculoskeletal physiotherapists [8,11], there are parallel concerns about this model of care, notably expressed from physiotherapists around the quality and safety of care [26,27]. Sustained investment in life-long training is a pre-requisite for a fit-for-purpose healthcare workforce [28].

GPs and physiotherapists expressed sentiments that pointed towards a form of musculoskeletal competency competition. When discussing musculoskeletal expertise and competency, physiotherapists reflected that while GPs might be safe in their management of patients with musculoskeletal disorders, they were effective mainly in terms of throughput but without necessarily offering effective solutions to the presenting problem:

(…) I do not see that the GP model in its current format is serves its intended purpose, it does not support safety and effectiveness. Some might say that they are very effective, but the question is whether they are effective at solving the problems in front of them. And I don’t necessarily think they are, as I see it. They have too little time, there are too many things to be done. (Manual Therapist)

Because I notice that when they are seen quickly and have pain or difficulty with movement, I give them some simple advice and they then feel reassured, start moving and get better. Or they may not get better, but they dare to move, and they function better. Whereas when they see the doctor, they might be told to take some painkillers. And then they don’t get better. And then they are referred for an orthopaedic assessment. And then they return to me after a few months. That’s what often happens. (Physiotherapist)

A GP expressed that the frequency of encountering common musculoskeletal disorders enabled GPs to build up competence:

We think we are good at everyday issues. (…) The most common things are the most common, I usually say. After all, there are a lot of shoulder and elbow tendinopathies, and meniscus degenerations in knees, and arthrosis, lumbago. So, we become good at those. (GP)

Another GP opined that GPs lack musculoskeletal competency and linked this to medical training:

So they’ve done some studies on, for example, final-year medical students, they’ve done some studies on interns, and LIS doctors [First-level internship of postgraduate medical specialisation, equivalent to UK’s Foundation Program] (…) this whole pathway, what about their musculoskeletal knowledge? They are all uncertain, everyone. They feel most uncertain when it comes to musculoskeletal problems. (GP)

#### Task preferences

Several GPs mentioned the introduction of direct access to midwives as an illustration of task shifting that had unintended consequences, such as losing clinical contact and becoming merely a sickness certificate issuer for this group of patients. One GP talked about the potential benefits of multidisciplinary working but also highlighted a fear of losing access to the musculoskeletal patient group and thereby deskilling in this area:

As GPs we are fortunate to have the broad scope that we have (…), but in another field, gynaecology and perinatal care, in some areas we see GPs hardly deal with it anymore, midwives have taken over. And if you lose the exposure, you lose the competence. (…) If everything related to musculoskeletal is taken over, if we no longer deal with it, I’d be sceptical about that. Because then the GPs would become really poor at it. (…) Because I see among my colleagues that if you have a musculoskeletal interest, you can be a good GP for many of these patients. But those who don’t have an interest in it (…) if you then take away their musculoskeletal patients, they will deskill in this area. (GP)

A physiotherapist recounted a conversation with a GP colleague, where the same fear of deskilling was expressed:

But I had a very brief conversation (…) with one of our GPs. (…) He said, ‘I don’t want to give up that role, or musculoskeletal patients, because that’s what maintains my musculoskeletal competence.’ So he didn’t want fewer patients. Also, they were nice patients to treat. (Physiotherapist)

Another GP thought that for task shifting to offer a benefit for GPs, the physiotherapists also had to take responsibility for what was seen as more challenging patients:

I think you have to examine this in depth, to ensure it serves both parties. It’s not easy to simply say it provides relief. In what way does it provide relief? For which patients? Those who are straightforward, or those who have chronic problems? How are these therapists supposed to help those with chronic problems? Who will be responsible for them? That also falls under musculoskeletal problems, right? (GP)

These deliberations around financial matters, competence, and task preferences point toward a dilemma of street-level accountability. If task shifting in this context is proposed as a way of increasing professionalism to solve a problem in primary care, we should consider Lipsky’s words of caution when he argued that whilst professions embody a service ideal in theory, there might exist a gap between this theoretical ideal and the professional service orientation in practice [18]. The professional ethos of acting in a way that bring advantages to patients is fundamental to healthcare professions, but is tested to breaking point when faced with the clinical reality of making choices that might result in disadvantages to yourself. In turn, this presents a challenge to policy makers: how to govern a field of healthcare professionals that vacillate between being a rational actor and protector of the public purse on the one hand, to at times abandoning their social purpose on the other.

It is likely that the way in which GPs and physiotherapists inhabit their role is neither uniform nor consistent over time. As street-level bureaucrats they inhabit a complex moral ecosystem that can be opaque to hierarchical scrutiny and control, an ecosystem within which clinicians must find a delicate equilibrium between a competing array of normative pulls, whilst navigating their approach to core demands of street-level work: people processing, service provision, and regulation [29]. Therefore, perhaps the best a GP or physiotherapist can hope to offer is a reluctant embrace of the contradictions of advocacy and accountability.

### Historical hegemony and established practice challenged by the emergence of task shifting

We discovered an apparent contradiction in the professional relationship between GPs and physiotherapists. On the one hand participants expressed opinions about how they appreciate a close working relationship with the other profession, about how co-location would give opportunities and make collaboration easier, but simultaneously they suggested that history and hegemony maintain the status quo and laid some blame at the door of professional organisations for resisting change. A physiotherapist extolled the benefits of having a low threshold for communication with GPs, and a GP pointed towards lack of resources as the barrier to his wish of setting up a co-located multidisciplinary team:

So the ongoing dialogue we have, that’s what makes it such a good collaboration. And that they know that I’m here if there’s anything they’re unsure about, and conversely, that there’s a low threshold for getting in touch, and they take the time to do so. (Physiotherapist)

The importance of multidisciplinarity and co-location, i.e. multidisciplinary collaboration between professions, I think that is the most important thing. (…) If I had (…) unlimited resources to be able to rent a location and create a team, I think it would become an extremely exciting professional environment. (GP)

A manual therapist highlighted the different attitudes between GPs and the Norwegian Medical Association about extending the right to issue sickness certificates to manual therapists:

I note, for example, that the GPs and the Medical Association probably have far too much power in wanting to maintain control just for the sake of having control. When manual therapists were given the right to issue sickness certificates, there was also, at least centrally in the Medical Association, a lot of resistance. It may be that cooperation works well locally, while there is opposition centrally. (Manual Therapist)

The dichotomy of being both friendly neighbours and hard-nosed competitors might indicate differences of opinions, but this could also represent how the context of vested interest and power influenced the interviews. A romantic perspective on interviews is based on the idea that the participant is sincere and selfless, willing to share their experiences and expertise for the benefit of the interviewer and the research project [24]. This viewpoint presents the interviewee as a person acting in the interest of science and of the researcher doing the interview. However, it is also possible to assume that the interviewee is acting in the interest of the profession he represents, seeking to protect or enhance the profession’s reputation and legitimacy, thereby becoming politically aware and a politically motivated actor [24]. There is not necessarily a conflict between honesty and treating the interview as political action in this way, but it might influence the way in which opinions are framed, for example through the selection of anecdotes or stories shared with the interviewer. It is also possible that the interviewees are influenced by uncertainties about how the interviewer will use the empirical material collected. This might well have affected GPs and physiotherapists differently; GPs might have seen the interviewer (a physiotherapist) as a potential threat whilst the physiotherapists might have considered the interviewer an ally in struggle for legitimacy and influence of their profession. Independent of how the participants experienced this, common to the two professions is the ideal of offering value to their patients, which we will discuss next.

#### Value-based healthcare and patient safety

The concept of value-based healthcare is emerging as a description of the transparent use of available resources to improve health outcomes, and the core contributions of health systems to societal wellbeing are suggested as health improvement, responsiveness, financial protection, equity, and efficiency [30]. The different actors within health systems can contribute to achieving these ambitions, but they might also offer resistance. From the aim that primary care contributes to societal wellbeing logically follows the reasonable expectation that healthcare professionals working in primary care embrace (new) models of working that promote value-based healthcare. GPs and physiotherapists naturally have a preoccupation with the financial sustainability of their business, and the health system relies on them to create value by providing services that generate health benefits. The European Commission Expert Panel on Effective Ways of Investing in Health propose four pillars of value underpinning the concept of value-based healthcare: technical, allocative, personal, and societal [30]. We will look at how the first two of these might be used to illustrate possible inefficiencies regarding how patients presenting with musculoskeletal disorders are currently managed in Norwegian primary care.

Technical value refers to the achievement of best possible outcomes within the limits of available resources [30]. When participants describe physiotherapy practice where there is a tradition to have regular treatment that continues for several years, or where the treatment diverges from best practice or evidence-based care, this represents practice that is technically inefficient. In turn this adds to waiting lists with the result that the needs of patients with acute presentations cannot be met in a timely manner.

I have often been one of few manual therapists at a physiotherapy clinic where there might be a tradition of providing regular treatment over many, many years. So there is unfortunately a low turnover [of patients] in many of the practices. Consequently, there are no opportunities to book in acute patients, as we would want. (Manual Therapist)

Where there is spare capacity, the financial imperative might also lead to this questionable behaviour described by a manual therapist:

It could also be that if you are in a situation where there is no waiting list, it may be tempting for someone to rebook a patient just to fill the list. (Manual Therapist)

Allocative value refers to the equitable distribution of resources across all patient groups [30]. When our participants discuss how patients are managed it seems that a degree of allocative inefficiency arises, through the provision of multiple assessments (by different professionals) for the same complaint or from the unnecessary request for further investigations. This can result in both unnecessary burden of disease for the patient and in excessive healthcare expenditure. A GP described a situation where demanding patients might influence clinical decision making in a negative direction:

Younger GPs are a bit more schooled on staying calm under pressure and choosing wisely, and to limit the use of diagnostic imaging, whilst I think a lot of my older colleagues are a bit more like, that they refer on too quickly out of habit. And patients are very demanding. They have great faith in diagnostic imaging. (GP)

Whilst it might be tempting to describe patients as over-cautious over-users of limited healthcare resources, it is worth considering patient safety in this context. If task shifting is considered a strategy for the introduction of a new model of care, the possible impact on patient safety – the prevention of errors and adverse effects to patients associated with healthcare [31] – is a key concern [32]. If a patient was to see a physiotherapist instead of a GP, where would the responsibility rest if unfortunate sequelae were to develop because of a missed red flag? When it comes to fulfilling the primary care gatekeeper role with its weighty responsibilities, GPs typically have education and training beyond that of physiotherapists. Finding the balance between avoiding overtreatment and ensuring patient safety is critical when implementing policies that include task shifting.

A GP suggested that GPs are responsible for overtreatment and that they offer interventions that do not offer value to the patient or to society:

The general conditions? Well, I think it’s actually very good business. It may have been a bit too good a business, in that we’re doing too much, maybe. That what we’re doing is providing overtreatment. And that we don’t always reflect on what we’re doing. So that, yes, maybe we’re doing things that don’t give any benefit to the patient or society over time. (…) You get the same pay by just carrying on offering something that may not have any proven effect. (GP)

A manual therapist also reported examples of overtreatment in physiotherapy clinics, where patients with long-term symptoms receiving regular treatment without an endpoint contributed to long waiting lists. Two other GPs also highlighted a lack of alignment with the principles of Choosing Wisely [33]:

The physiotherapists offer lots of things that are not evidence based, and so do we at the GP surgery. (GP)

The link between Choosing Wisely and the tariff system, for example, there is no link. (GP)

Through the lens of street-level bureaucracy theory these examples of inconsistencies with the principles of Choosing Wisely and evidence-based practice describe behaviours and intentions where clinicians see themselves as more than cogs in the wheels of the healthcare system; they are also moral agents that must make rational choices and exercise considerable discretion when managing patients. These choices and discretions reflect the balancing act of trying to fill different roles that of patient advocate, of health service gatekeeper, of homo economicus.

#### A case for change

Multidisciplinary teams are needed to deliver high-quality health services, and such multidisciplinary teams enable task shifting to allow each profession to work at the top of their licence [28]. In Norwegian primary care the management of patients with musculoskeletal disorders remains fragmented and to an extent reflects a supply-driven system. The organisation of care where GPs and physiotherapists typically operate from separate locations might prevent effective collaboration and to an extent, person-centred care. The recent report on the Norwegian GP system reported that a change to more multidisciplinary working in primary care, to include for example nurses, psychologists and physiotherapists, would lead to increased costs for municipalities [4] and this presents a challenge to the central priority of task shifting from GPs to other healthcare professionals.

If history has taught us anything, it is that the direction of change is not pre-determined. In the realm of healthcare delivery models, various discourses - shaped by socio-cultural changes (such as to the patient role) and political processes (such as the commercial nature of work) – set the stage for transformation, and task shifting emerges as a central actor in this setting. Foucault’s concept of genealogy highlights how discourses give rise to one another, often beyond rational connections [20]. For Foucault, power is an unstable network flowing in all directions from every point at once, where we are all exerting power on our surroundings through social, cultural, and professional norms, through encouragement or discouragement of certain behaviours we approve or disapprove of, and through social and economic behaviour. The accumulative force of this power affects the direction of change. We are constantly defining and redefining what is normal, who should be taken seriously and listened to. This makes power a diffuse entity, where we simultaneously shape and are shaped by both friends and colleagues, as well as professionals and governments. The implementation of the task shifting we examine might lead to a blurring of the lines between the historical GP hegemony and emerging direct access model of care to other healthcare professionals.

Foucault’s notion of biopower can be described as the power over life and how lives may be managed at both the individual and group levels [34]. Biopower is harnessed through scientific discourse and cultural norms, allowing those who control the dominant narrative to hold the reins of power, as they control the parameters, the language, the concepts, what is worth spending efforts on. However, the historical power imbalance between GPs, physiotherapists, and laypeople is gradually diminishing due to factors such as improved information accessibility. This might over time change the current set of dominant narratives, which will inexorably be replaced by another. However, as Foucault pointed out, a replacement narrative may – or may not – be more tolerable than its predecessor. A current dominant narrative is the concept of continuity of care.

Continuity of care, where patients have a consistent relationship with their GP, has been associated with several positive healthcare outcomes [35]. At the same time, there appears to be no difference between the number of consultations per year or in emergency care visits between those registered with a GP and those who are not [36]. Further, the benefits of having a named GP might be less clear for the patient who attends once a year compared to the patient who is seen by the GP several times a month [37]. The ageing population and increase in complexity presenting in primary care necessitate improved coordination and teamwork and challenge the model of GPs single-handedly managing their list of patients from cradle to grave, and we should explore how the continuity-of-care concept could be maintained, or even improved, by multidisciplinary collaboration. Multidisciplinary collaboration in primary care is considered crucial to deliver person-centred care [38], and a shared work location is reported as an important element of interprofessional teamwork [39]. Patients are both users and choosers of healthcare and if we want primary care to be person-centred, we should recognise that both the professional discipline and role of the healthcare provider should change according to the needs of the patient.

Our study supports previous research on the need for change in musculoskeletal healthcare, and we contribute new knowledge about different processes that act as drivers of or barriers to change. In their roles as street-level bureaucrats GPs and physiotherapists absorb a significant amount of public resources, and these bureaucrats, as suggested by Lipsky, come to represent the hope of society for a healthy balance between the delivery of public services and a reasonable burden of public spending [18].

#### Limitations

We anticipated that recruiting 6-10 GPs and the same number of physiotherapists would be sufficient to explore the aims of the study. This estimate was based on the rationale developed by Malterud et al. [40] who proposed the concept of *information power* to guide adequate sample size for qualitative studies. We believe that our participants held large information power and that therefore a relatively lower *N* was needed, based on these elements of information and their relevant dimensions:

- The aim of the study: Our study has narrow and focused aims.
- Sample specificity: Our participants have extensive experience and knowledge about the topic and the sample specificity is therefore dense.
- Use of established theory: Our study is informed by two specific theoretical perspectives (Lipsky and Foucault).
- Quality of dialogue: We anticipated that the quality of the dialogue would be strong, as the lead researcher (KCL) holds significant background knowledge about the topic and has experience of carrying out similar interviews.
- Analysis strategy: We chose an analytical approach [24] suitable for in-depth analysis of narratives, where the intention was to explore selected aspects relevant to the study aims.

Whilst we ended up recruiting one less GP than originally intended, we believe that the participants held sufficient information power to inform our study.

It is important to point out that our sample of participants is neither comprehensive nor necessarily representative of the GP or physiotherapy professions. The five GP participants all carried out their clinical practice in Centrality Index 2 areas, and their views and experiences might differ significantly from colleagues who work in other areas. The inclusion of physiotherapists from all Centrality Index areas (1–6) gives a higher level of possibility that any geographical influence was captured in the interviews.

Further, it is possible that the interviewer’s professional background might have affected the GPs and physiotherapists differently. The interviewer (KCL) is a physiotherapist with a Manuel Therapy qualification and the GPs’ contributions might have been cloaked in a veil of ‘this is what I think he would like to hear’. This confounding effect might have been different for the participants with a similar professional background, where an innate understanding of ‘we’re on the same side here’ might have prevailed. The interviewer had distributed information about the development of a musculoskeletal core capabilities framework [41] and wanted the interviewees to be introduced to the First Contact Practice model where physiotherapists take on a particular role, and this might have influenced how opinions were formulated and shared with the interviewer. It is also possible that the interviewer’s background might have swayed participants into agreeing to take part.

Finally, the chosen analytical framework diverges from the traditional approach of looking for patterns and themes within the empirical material. Our approach instead explored the dialectic between patterns and fragmentations, our analysis is shaped by preunderstandings and theoretical ideas with an acknowledgement that opinions are socially constructed and situated, and we attempted to challenge conventional views to offer insights that might reveal the unfamiliar or unexpected in a familiar social domain.

## Conclusion

In this study we have developed theoretical reflections that probe possible roots and causes of the status quo in primary care, and explored an apparent reluctant embrace of the necessity for change. Through our analyses and interpretations of interviews of GPs and physiotherapists we have identified novel angles of enquiry about changes to primary care in Norway. The current iteration of primary care is not fixed, and both the speed and direction of change are influenced by many forces. Change might be achieved through changing systems, structure, and policy, and through the clinical practice and initiatives by individual practitioners. We offer interpretations that can work at the level of recognition, which may help to identify current challenges and opportunities as the efforts to improve models of care continue.

Any development and implementation of a new model of care, such as the First Contact Practice model to build musculoskeletal capacity in primary care, would necessitate the creation of an efficient, effective, and functional network between the stakeholders involved. Whilst healthcare system transformations are slow, over time change is inevitable. We do not know whether the specific model of task shifting explored in this study is a destination we should aim for. Nevertheless, it can be useful to explore the direction of travel even without being set on a destination. This study offers some theoretical reflections that might contribute to the current debate around task shifting in musculoskeletal care in Norwegian primary care.

## Supplementary Material

Appendix 1 Interview guide.docx
