# Supplementary material for: Task shifting for musculoskeletal disorders in Norwegian primary care: a qualitative interview study of general practitioners and specialist musculoskeletal physiotherapists
Source: Scand J Prim Health Care. 2024 Aug 2;43(1):13–23. doi: 10.1080/02813432.2024.2384043 (PMC11834803; doi:10.1080/02813432.2024.2384043)
Supplement: Appendix 1 Interview guide.docx [file IPRI_A_2384043_SM3842.docx]

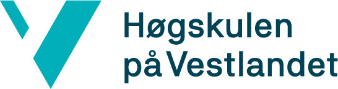
 V1.7_05.10.2023 (final)

**INTERVIEW GUIDE**

**Task shifting for musculoskeletal disorders in Norwegian primary care: a qualitative interview study of general practitioners and specialist musculoskeletal physiotherapists.**

## INTRODUCTION

1. Explain the purpose of the interview and anticipated duration (60 minutes), confirm that the participant consents to audio recording of the interview, and give brief outline of the topics to be explored.
2. Ask participant whether there are any questions relating to the Participant Information Sheet and clarify consent.

## PARTICIPANT INFORMATION

1. Year of professional qualification.
2. Any specialisation within musculoskeletal health?
3. Describe your place of work.
   Possible follow-up questions:
   1. Size of practice; other professions co-located; waiting lists (for physiotherapists); list size (for GPs); patient demographics.
4. How would you describe the way you approach the primary contact role to another healthcare professional (from another profession)?
5. What – if any – are your experiences of inter-professional collaboration and/or task shifting within your professional role?

## BACKGROUND

1. How would you describe the current primary care provision for people with a suspected musculoskeletal disorder?
   Possible follow-up questions:
   1. What works well; what is missing; what should be available?
   2. Do you have any thoughts about why things are as they are? What do think about the responsibilities of different stakeholders (patients, GPs, physiotherapists, politicians, others)?
   3. Do you have any suggestions to how primary care can be improved for patients with musculoskeletal disorders?
   4. How would you describe the financial frameworks for GPs/physiotherapists working in primary care?
   5. Does the financial model constitute a challenge to providing evidence-based practice?
2. How well is multi-disciplinary working integrated in Norwegian primary care?
   Possible follow-up questions:
   1. Does this differ from secondary care? If so, why?
   2. How can MDT working be improved in primary care?
3. What are your thoughts around the issuing of sickness certificates, specifically around return-to-work aspects?
   Possible follow-up questions:
   1. Do you think it matters if the sickness certificate is issued by a GP or a manual therapist?
   2. What, if any, effect could an increase in MDT working in primary care have on the issuing of sickness certificates?

## THE fcp MODEL OF CARE

1. Short explanation of the FCP model of care. Ask if participant engaged with any of the FCP information signposted to in the Participant Information Pack, and if they have any questions about the English FCP model.
2. Do you think that a physiotherapist in the FCP role could be a safe gatekeeper for patients who present in primary care with a suspected, but undiagnosed, musculoskeletal disorder?
   Possible follow-up questions:
   1. Do you think the FCP model could lead to increased risk for patients, for example through misdiagnosis?
   2. Do you think the FCP model would have an effect on resource utilisation, such as referral for imaging or to specialist care?

## tHOUGHTS ABOUT AN FCP model OF CARE iN norWAY

1. What are your thoughts about the possibility of introducing task shifting - similar to the FCP model of care - in Norway?
   Possible follow-up questions:
   1. Do you think physiotherapists/manual therapists can reduce the demands on GPs by taking on more of the patients with musculoskeletal complaints?
   2. What would be the possible consequences of this?
2. Patients have direct access to physiotherapists (since 2018) and manual therapists (since 2006), do they have the required competencies and capabilities for this responsibility?
   Possible follow-up questions:
   1. Why/why not?
   2. Would a physiotherapist also require a manual therapy qualification to be an FCP in Norway?
3. Do GPs have the necessary competencies and capabilities to be safe and effective gatekeepers for patients who present in primary care with a suspected, but undiagnosed, musculoskeletal disorder?
4. What impact, if any, do you think an introduction of the FCP model would have on GPs, physiotherapists, manual therapists, and patients in Norway?
5. In England, the FCP model is financed through the Additional Roles Reimbursement Scheme. Do you have any thoughts on how an FCP model could be financed in Norway?
6. A key aspect of the FCP model is the co-location of GPs and physiotherapists, to enable peer support. Would this be practically possible in Norway?
   Possible follow-up question:
   1. What are the possible enablers and barriers?
7. Do you have a solution on what is commonly referred to as the ‘GP crisis’ in Norway?
8. What do you think about this statement about the reason for some of the problems facing the health service: ‘Patients are empowered to have rights, rights that health care professionals experience that they are unable to fulfil’.
9. Our preliminary analyses suggest that GPs, physiotherapists, and manual therapists take on several roles (patient advocate, gatekeeper, homo economicus), and must address different forces when balancing these. What are your thoughts about this?
10. Another preliminary finding is that established hierarchy and hegemony has an impact on attitudes to task shifting, sometimes contrary to also expressing support for the underlying principles. What are your thoughts about this?

## avslutning

1. It has been more difficult to recruit GPs to take part in this study than physiotherapists and manual therapists. What do you think could be the reasons for this?
2. What do you think are the two most important aspects of our conversation?
3. Do you have any suggestions about questions I should ask the next interviewee? What would your answer be to this?
4. Is there anything else you want to add?
5. Can we contact you again if necessary?

Explain that the audio recording of the interview will be transcribed and de-identified within four weeks, and subsequently deleted, and that informed consent can be withdrawn at any time until this has taken place.

Invite the interviewee to get in touch if he/she wants to add anything that might be useful.
